# Thermo-Analytical and Compatibility Study with Mechanistic Explanation of Degradation Kinetics of Ambroxol Hydrochloride Tablets under Non-Isothermal Conditions

**DOI:** 10.3390/pharmaceutics13111910

**Published:** 2021-11-11

**Authors:** Dijana Jelić, Snežana Papović, Milan Vraneš, Slobodan Gadžurić, Silvia Berto, Eugenio Alladio, Dragana Gajić, Bojan Janković

**Affiliations:** 1 Chemistry Department, Faculty of Sciences and Mathematics, University of Banja Luka, 78 000 Banja Luka, Bosnia and Herzegovina; dragana.milisavic@pmf.unibl.org; 2 Department of Chemistry, Biochemistry and Environmental Protection, Faculty of Sciences, University of Novi Sad, 21 000 Novi Sad, Serbia; snezana.papovic@dh.uns.ac.rs (S.P.); milan.vranes@dh.uns.ac.rs (M.V.); slobodan.gadzuric@dh.unsac.rs (S.G.); 3 Department of Chemistry, University of Turin, 10125 Turin, Italy; silvia.berto@unito.it (S.B.); eugenio.alladio@unito.it (E.A.); 4 Department of Physical Chemistry, “Vinča” Institute of Nuclear Sciences—National Institute of the Republic of Serbia, University of Belgrade, 11 000 Belgrade, Serbia; bojan.jankovic@vin.bg.ac.rs

**Keywords:** ambroxol hydrochloride, thermal stability, compatibility, chemometrics

## Abstract

Ambroxol hydrochloride (AMB), used as a broncho secretolytic and an expectorant drug, is a semi-synthetic derivative of vasicine obtained from the Indian shrub *Adhatoda vasica*. It is a metabolic product of bromhexine. The paper provides comprehensive and detailed research on ambroxol hydrochloride, gives information on thermal stability, the mechanism of AMB degradation, and data of practical interest for optimization of formulation that contains AMB as an active compound. Investigation on pure AMB and in commercial formulation Flavamed^®^ tablet (FT), which contains AMB as an active compound, was performed systematically using thermal and spectroscopic methods, along with a sophisticated and practical statistical approach. AMB proved to be a heat-stable and humidity-sensitive drug. For its successful formulation, special attention should be addressed to excipients since it was found that polyvinyl pyrrolidone and Mg stearate affect the thermal stability of AMB. At the same time, lactose monohydrate contributes to faster degradation of AMB and change in decomposition mechanism. It was found that the n-th order kinetic model mechanistically best describes the decomposition process of pure AMB and in Flavamed^®^ tablets.

## 1. Introduction

Many drugs are intended for oral administration, and the most used are solid dosage forms such as tablets, capsules, and powders. To develop a stable, safe and effective final drug product, it is essential to characterize the physicochemical properties and assess its compatibility with excipients during the processing and storage [1,2]. Excipients in the solid dosage forms can contain relatively high water content but in different percentages. For example, the equilibrium moisture content of polyvinyl pyrrolidone, PVP, is about 28% at 75% relative humidity [3]. One should pay attention that such excipients can affect and increase the degradation rate of active drug ingredients. The moisture level can affect the stability depending on how strongly it is bound and whether it can contact the active drug. Heat and moisture accelerate most reactions, even if moisture is not involved in the reaction scheme since moisture brings molecules closer together, and heat always increases the reaction rate [4].

Kinetic information is crucial for evaluating the times and temperatures associated with the processing service lifetimes and storage of materials. Thermo-analytical (TA) techniques have been used to rapidly evaluate the purity, degradation kinetics, and physical property of drugs [5,6,7,8,9,10,11]. A typical application is the use of non-isothermal thermogravimetric (TG) runs for estimating the thermal stability and shelf-life of a material (drug) at a specific temperature [12,13]. Moreover, these techniques alerted compatibility problems and indicated the most favorable directions for a successful formulation [14,15]. In the present study, we focused our attention on the thermal stability of the commercial pharmaceutical tablet formulation (Flavamed^®^) (FT), in which ambroxol hydrochloride (AMB) is an active component.

**Figure 1:**
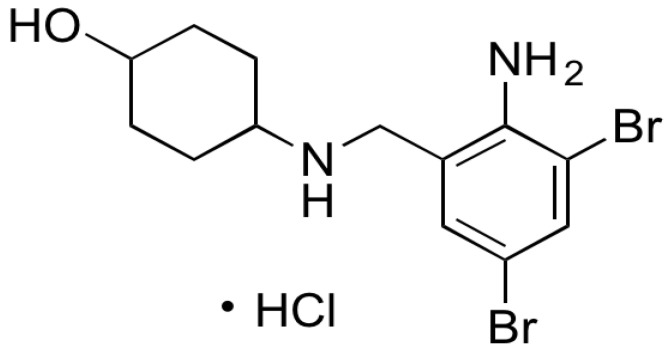
The structural formula of ambroxol hydrochloride.

AMB is chemically (1s,4s)-4-((2-amino-3,5-dibromocyclohexyl)methylamino)cyclohexanol hydrochloride (Figure 1). Its melting point is 235 to 240 °C [16]. Ambroxol hydrochloride is a semi-synthetic derivative of vasicine obtained from the Indian shrub *Adhatoda vasica*. It is a metabolic product of bromhexine. It is used as a broncho secretolytic and an expectorant drug [16,17]. It stimulates the transportation of the viscous secretions in the respiratory organs and reduces the stand stillness of the secretions. AMB is a clinically proven systemically active mucolytic agent. When administered orally, the onset of action occurs after about 30 min [16]. Ambroxol hydrochloride is completely absorbed after oral administration [16]. Ambroxol is changed into various inactive metabolites that are mainly eliminated as water-soluble conjugates. After oral administration, 85% of the active substance is eliminated in the urine. Less than 10% is eliminated in the form of unchanged ambroxol [16].

According to Pharmacovigilance Risk Assessment Committee (PRAC) report from 2015, mucolytic drug AMB, including ambroxol-containing products indicated for secretolythic therapy, infant respiratory distress syndrome, and in the prevention of postoperative complications, has been used by more than four million people in the last decade (2004–2013) and AMB is currently used in the 50 countries worldwide [18]. Different formulations of ambroxol are available on the market, including the formulation of extended-release (ER) capsules, pastilles, and syrups for adult and pediatric use. Reported efficacy was comparable among formulations with minor differences in favor of the pediatric syrup [19].

Ambroxol hydrochloride molecule character is unstable under an oxygen atmosphere or alkaline environment, which can contribute to the decomposing process and might reduce the safety of AMB [20]. There is no detailed and comprehensive research on ambroxol hydrochloride thermal decomposition and its stability in the current literature. Podbukowska et al. reported on some physicochemical properties of AMB: solubility, acidity constant determination, and thermophysical data [21]. Caira et al. investigated the thermal and structural properties of ambroxol polymorphs [22]. Mo et al. investigated compatibility between two drugs, AMB and omeprazole, in a mixture by DSC technique [23] and proved that omeprazole becomes less thermal stable in the presence of ambroxol hydrochloride. In addition, combining AMB with some other drugs, such as terbutaline, improved the clinical efficiency of ambroxol [24]. Due to AMB’s poor water solubility, α and β cyclodextrins were evaluated as potential guest molecules for AMB. Ambroxol hydrochloride membrane-coated matrix tablets were successfully prepared using hydroxypropyl methylcellulose (HPMC). The viscosity and the amount of HPMC were significant for acquiring zero-order release kinetics [25]. Kardos et al. reported on the characterization of differential patient profiles and therapeutic responses of pharmacy customers for four ambroxol formulations [19]. Generally, the kinetics of ambroxol hydrochloride seems to be rather poorly studied.

Combining thermal methods (simultaneously thermogravimetric analysis and differential scanning calorimetry, TG-DSC) with mass spectrometry (MS), along with chemical kinetics and chemometrics tool, we provided a specific kinetic approach in revealing a detailed mechanistic feature of AMB and FT thermal degradation under dynamic (non-isothermal) conditions in N_2_ atmosphere. The choice of isothermal (T = const.) or non-isothermal (β = dT/dt) experiments depends on the requirements of the study, whether one wishes to study reaction kinetics over wide temperature ranges (i.e., up to the melting) or if a narrow range is enough. Various models included in contemporary kinetic software were evaluated and compared (fitted) to determine the importance of the different degradation pathways.

Furthermore, in this paper, special attention was given to the role of presented excipients for the stability determination of the dosage form and the evaluation of shelf-life values to prepare the ideal optimization procedure for the storage of pharmaceutical preparations [26,27]. It should be noted that the small pharmaceutical molecules and their reactions in the solid state have attracted the attention of scientists. With the increasing use of small organic molecules as pharmaceuticals, it is crucial to understand the solid-state behavior of compounds. Compatibility studies between active compounds and excipients in Flavamed^®^ formulation were conducted systematically through thermal and spectroscopic characterization, and chemometric tools analyzed the data.

Therefore, this paper, combining exhaustive experimental and newfangled statistical methods, presents an overall contribution to degradation mechanism, kinetics, thermal stability, compatibility assessment of AMB, and its rational drug formulation. Considering widespread usage of AMB mucolytic agents for chronic bronchitis or chronic obstructive pulmonary diseases, detailed analysis such as this one might provide paths for the upgraded formulation of ambroxol hydrochloride-containing drugs [28].

### 1.1. Theoretical Basis for Data Analysis

#### A Kinetic Analysis—Theoretical Approach

A typical starting point in the kinetic analysis based on thermal analysis data is Equation (1):

(1) $$\frac{d\alpha }{dt}=kf\left(\alpha \right)=Ae^{-E/RT}f(\alpha )$$

where *α* is the conversion degree, *t* is time, *f(α)* is a mathematical expression in which form is dependent on the reaction mechanism and *k* = *Ae*^−*E*/*RT*^, where *A* is the pre-exponential factor, *E* is the apparent activation energy, *R* is the gas constant, and *T* is the thermodynamic temperature. The mass loss data from the TG curve can be transformed into conversion degree (*α*) by means of the equation:

(2) $$\alpha =\frac{m_{0}-m_{t}}{m_{0}-m_{f}}$$

where *m_t_* represents the mass of the sample at arbitrary time *t* (or the temperature *T*), whereas *m*_0_ and *m_f_* are the mass of the sample at the beginning and the end of the process, respectively [29].

Such transformed TG data are easily operated by the proper kinetics software, which in return provide information on the kinetic scheme and corresponding kinetic triplet (*E*, ln*A*, and *f*(*α*)). The exact determination of kinetic parameters is based on multiple scan methods, which require the measurements at different heating rates and use the data sampled at joint conversion degrees (isoconversional, model-free methods). Kinetics software, Kinetics 2015, uses various simple linear regression methods to determine initial guesses for non-linear regression of various kinetic models [29]. All the linear regression methods in Kinetics 2015 are variations of well-known isoconversional methods. The basic Friedman equation, isoconversional model, solves the simple nth-order reaction rate equation for the rate constant, *k*, as [30]:

(3) $$\frac{d\alpha }{dt}=k{\left(1-\alpha \right)}^{n},$$

where *dα*/*dt* is the process rate, *α* is the conversion fraction or fraction reacted, and *n* is the reaction order. For any set of experiments with different, arbitrary thermal histories, a plot of ln(*dα*/*dt*) versus 1/*T* has a slope of −*E*/*R* and an intercept ln[*A*(1 − *α*)*^n^*] (where *E* represents activation energy (J·mol^−1^), and *A* is the pre-exponential (frequency) factor (s^−1^)). If one truly has an nth-order reaction with n not equal to unity, it is theoretically possible to obtain constant apparent activation energy and extract a reaction order, but this rarely occurs in practical cases.

The expanded Friedman method, given by Equation (4), is used more frequently as [31]:

(4) $$\ln\left(\frac{d\alpha }{dt_{\alpha }}\right)=-\frac{E_{\alpha }}{RT_{\alpha }}+\ln\{A_{\alpha }f(\alpha )\}$$

Here *t_α_*, *T_α_*, *E_α,_* and *A_α_* are time, temperature, apparent activation energy, and pre-exponential factor, respectively, at a given conversion degree *α*. The plot of ln(*dα*/*dt_α_*) vs. 1/*T_α_* enables to determine the slope −*E_α_*/*R* and the intercept with the vertical axis ln{*A_α_*·*f*(*α*)}. In addition, it is possible to predict parameters at any temperature. To employ the values *E_α_* and {*A_α_*·*f*(*α*)} extracted from Equation (4), introducing them into the Equation (3) modified in the following way:

(5) $$t_{\alpha }=\int_{0}^{t_{\alpha }}dt=\int_{0}^{\alpha }\frac{d\alpha }{\{A_{\alpha }f(\alpha )\}e^{E_{\alpha }/RT_{0}}}\;$$

No specification of the exact form of the reaction model *f*(*α*) is needed for this analysis, so Equations (3) and (4) form a complete base for all Friedman’s isoconversional kinetic measurements and predictions.

Introducing the heating rate, *β*, as a parameter that correlates time and temperature, Equation (1) can be alternatively written as:

(6) $$g(\alpha )=\frac{d\alpha }{f(\alpha )}=\int_{0}^{T_{0}}\frac{A}{\beta }\exp\left(-\frac{E}{RT}\right)dT\;$$

The Kinetics2015 non-linear regression analysis methods range from a single first-order reaction to various activation energy distribution models such as the discrete model. A first-order reaction model is the simplest and most commonly used kinetic model. It starts with the simple statement that the rate of disappearance is proportional to the amount present as *dα*/*dt* = *k*(1 − *α*), where (1 − *α*) represents the amount remaining, while k represents the rate constant ordinarily assumed to follow the Arrhenius law such as *k* = *Ae*^−*E*/*RT*^, where *R* is the universal gas constant. An nth-order reaction is a simple extension, and the basic rate equation was given by Equation (3). In many cases, the nth-order reaction is typically attached to solid decomposition, as a pseudo-nth-order reaction, because it does not have the classical meaning as the nth-order reaction in the gas phase or solution, in which the reaction rate is proportional to the probability of two reactants bumping into each other [32]. However, a more recent theoretical work has shown that the nth-order reaction model is functionally equivalent to having a gamma distribution of activation energies [32].

The nucleation-growth model, as a very useful kinetic model and often employed, which is practically an extended Prout–Tompkins model introduced by Burnham and Braun [33,34], can be expressed as:

(7) $$\frac{d\alpha }{dt}=k{\left(1-\alpha \right)}^{n}{\left(1-q(1-\alpha )\right)}^{m}\;$$

where *q* is an initiation parameter and *m* is a parameter related to the growth dimensionally or branching ratio, depending on whether the reaction is a solid-state or fluid-solid reaction. Burnham and Braun [33,34] set *q* value on 0.99, but it is user selectable. If *n* = 0 and *m* = 1, Equation (7) has the limit of the linear chain-branching model. If *n* = 1 and *m* = 0, it has the limit of a first-order reaction. If *n* = *m* = 1 it is the standard Prout–Tompkins model [34].

### 1.2. A Chemometrics Analysis—Theoretical Background

Principal component analysis, PCA is traditionally exploited to represent the acquired data into a new Cartesian graph (consisting of the calculated principal components (PCs)) by evaluating any cluster or similarities within the available samples and inferring the correlation among the measured variables [35,36]. PCA aims to remove the redundant and noisy information in the data by selecting a limited series of wavelengths, to obtain improved interpretability of the data. PCA computes new orthogonal and independent variables, named principal components, representing a linear combination of the original wavelengths aimed to reproduce the collected data matrix (X) information, but in an optimal and more interpretable way. This method decomposes the original matrix X into the product of two new matrixes, named T and P, plus a matrix of residuals E, as follows:

X(N,p) = T(N,c) × P(c,p) + E(N,p)(8)

where N is the number of samples (here, the different samples involving the compounds under analysis), p is the number of wavelengths (here collected within the IR range 400–4000 cm^−1^), and c represents the number of selected principal components. In particular, the first PC is oriented along the direction of the maximum variance. Subsequently, the second PC follows the maximum residual variance, chosen among the orthogonal directions considering the first component, etcetera. T matrix contains the samples’ new values (called scores) in the new multivariate space delimited by a c-number of PCs (i.e., scores plot). P matrix provides the variable vector coordinates (named loadings) in the new multivariate space (i.e., loadings plot). These linear combinations are computed by providing specific weighting coefficients to each wavelength to represent the influence between the original and new components. The loadings are the elements of the eigenvector of the variance-covariance matrix of the original X matrix. Each eigenvector has a corresponding eigenvalue that implies the amount of variance explained (EV) by each PC. In the present study, only the first two PCs were selected to account for a specific percentage (above 90% of cumulative explained variance, CEV) of the overall variance.

## 2. Materials and Methods

### 2.1. Materials

Flavamed^®^ packaging of tablets was provided by the international research-based pharmaceutical company Berlin-Chemie AG, Berlin, Germany (Menarini Group), Glienicker Weg 125, 12489 Berlin, Germany. Each tablet contains 30 mg of ambroxol hydrochloride (AMB). The external appearance of the tablet: white, round tablets with flat surfaces and faceted edges, with an embedded dividing line on one side. In addition to the active (drug) ingredient, the other excipients are as follows: (a) lactose monohydrate (filler in matrix tablet), (b) corn starch (disintegrant and binder in matrix tablet), (c) powdered cellulose (disintegrant in matrix tablet), (d) croscarmellose sodium (CCS) (FDA (U.S.—Food and Drug Administration) approved superdisintegrant), (e) povidone (polyvinylpyrrolidone, PVP) K30 (binder in matrix tablet) and (f) magnesium stearate (lubricant in matrix tablet). Ambroxol hydrochloride, a reference pharmaceutical standard, was purchased from Sigma-Aldrich, St. Louis, MO, USA.

### 2.2. Sample Preparations

Experimental samples for thermo-analytical measurements were the powder-formed tablets and pure pharmaceutical standard of AMB. Several tablets were crushed by a mechanical pestle, then minced and turned into white powders. The tablets were milled by such mechanical force so that we do not recognize any appearance of smooth surfaces that would possibly remain from the initial form of the same observations tablets. It can be pointed out that milling of the “particles” may generally change the surface characteristics by rendering the micro-surfaces more disordered or even amorphous. In the general case, particle shape and surface roughness affect the mechanical strength of the investigated Flavamed^®^ tablet.

### 2.3. Thermo-Analytical (TA) Measurements

The thermal stability of the samples was investigated by simultaneous non-isothermal (dynamic) thermogravimetric analysis (TGA) and differential scanning calorimetry (DSC) using a thermogravimetric analyzer by TA Instruments with SDT 2960 model device, capable for the simultaneous TGA-DSC analyses. TGA-DSC experiments were performed in the temperature range of 25–800 °C, under a dynamic atmosphere of nitrogen (N_2_ purity of 99.999 wt.%) at a flow rate of φ = 70 mL·min^−1^. Powder samples of about 5 mg were put into platinum crucibles at the heating rates of β = 5, 10, and 20 °C/min (AMB) and β = 5, 10, and 30 °C/min (FT). Heating of the samples was carried out under the linear heating regime, where there is a linear relationship between the increases in working temperature (within controlled conditions) with the time of operation (T = T_o_ + β·t, where T is the temperature (°C), T_o_ is the starting temperature of the non-isothermal experiment (ambient (room) temperature, T (°C)), β = dT/dt (the heating rate), and t is the time). Duplicate non-isothermal runs were made under similar conditions, and it was found that the experimental data overlapped with each other, indicating satisfactory reproducibility. Therefore, each run was duplicated to minimize the errors. The obtained thermo-analytical data at every considered heating rate (β) were directly transferred into the Kinetics2015 [29] software tool sheets for the corresponding calculation steps related to the kinetic studies.

### 2.4. TG-MS Measurements

Hyphenated TG-MS measurements were taken with the same thermal analyzer coupled online with Hiden Analytical HPR-20/QIC mass spectrometer. The sample (~2.5 mg) was placed in an open alumina pan. The measurements were carried out in argon atmosphere (flow rate: 100 cm^3^∙min^−1^), from room temperature to 450 °C, with a heating rate of 10 °C·min^−1^. Online coupling between the two parts was provided through a heated (T = 200 °C) 1 m silica capillary tube with an inner diameter of 0.15 mm. Selected ions between m/z = 1–110 were monitored through 30 channels in Multiple Ion Detection (MID) mode, with a measuring time of 0.5 s per channel. The mass spectrometer was operated in electron impact ionization mode with 70 eV electron energy power. The data collection for the MS measurements was performed as a function of time, which is proportional to the temperature of the sample. The RC RGA Analyzer and MAS soft manual set were used for the data collection.

### 2.5. Chemometrics Analysis

Principal components analysis (PCA) was employed to explore the collected data to extract the most interesting information from the samples analyzed by IR (infra-red) methodology [36,37]. The original data set consists of a matrix X containing 16 samples and 2528 wavelengths in 400–4000 cm^−1^. Samples were pre-processed involving the standard normal variate (SNV) approach to normalize the collected IR spectra before computing the PCA model [36]. The developed PCA model was calculated involving a 5-fold cross-validation approach with Venetian blinds sampling design. PCA models were computed using MATLAB software and the PLS Toolbox version 8.9.2 [37].

## 3. Results

### 3.1. Thermal Stability Features of Ambroxol Hydrochloride and Flavamed^®^ Tablets

**Figure 2:**
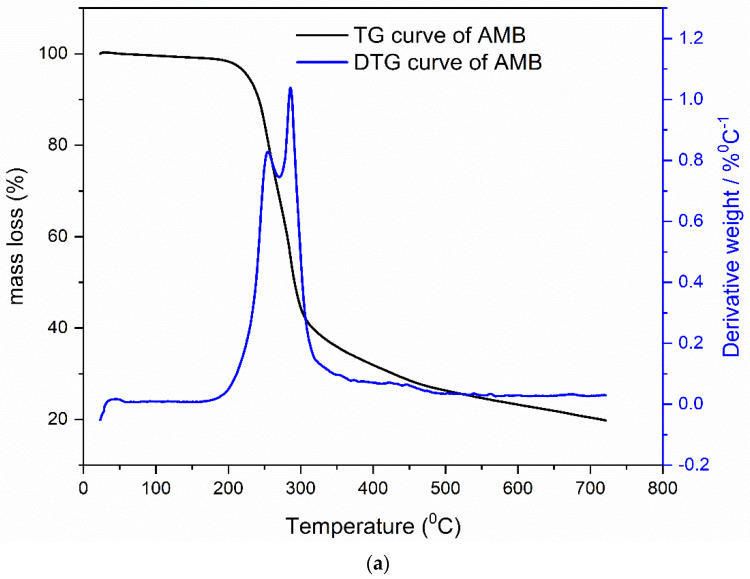

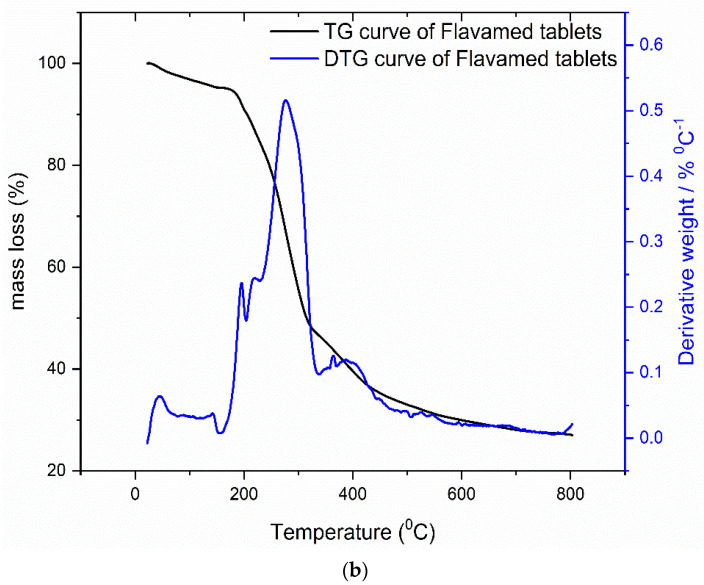
(**a**,**b**) The simultaneously TG/DTG curve of ambroxol hydrochloride and Flavamed^®^ tablet formulation at β = 10 °C/min in N_2_ atmosphere.

Figure 2a,b presents TA measurements of a pharmaceutical standard of ambroxol hydrochloride (AMB) and Flavamed^®^ tablet (FT), which contains ambroxol hydrochloride as an active compound. Thermal decomposition features presented by TG-DTG (thermogravimetry-derivative thermogravimetry) curves were conducted in an inert atmosphere (N_2_) at heating rate β = 10 °C/min where the entire thermal decomposition process of ambroxol hydrochloride and Flavamed^®^ tablets shows the very complex and multistep nature.

Pure AMB thermal decomposition is presented through three decomposition stages with corresponding T_i_ (initial temperature) and T_f_ (final temperature) for each stage: I stage = 30–175 °C, II stage = 175–280 °C, and III stage = 280–700 °C, respectively. AMB starts to melt around 175 °C, followed by the decomposition process through two rather inseparable stages (II and III) [18]. The thermal degradation of organic matter in the second stage corresponds with the mass loss of around 40%. In contrast, in the third stage, degradation products of the ambroxol drug were subjected to almost total decomposition, which ends at 700 °C. Flavamed^®^ tablets decomposition consists of four stages with following temperatures intervals: I stage = 30–160 °C, II stage = 160–262 °C, III stage = 262–318 and IV stage = 318–800 °C, respectively. The first stage presents the evaporation process. In the II and III stages, the degradation process of lactose monohydrate and AMB occurs. The latter one comprises multiply DTG peaks due to thermal decomposition of excipients mostly stable up to 300 °C, such as PVP [38], Mg stearate [39], corn starch [40], etc.

**Figure 3:**
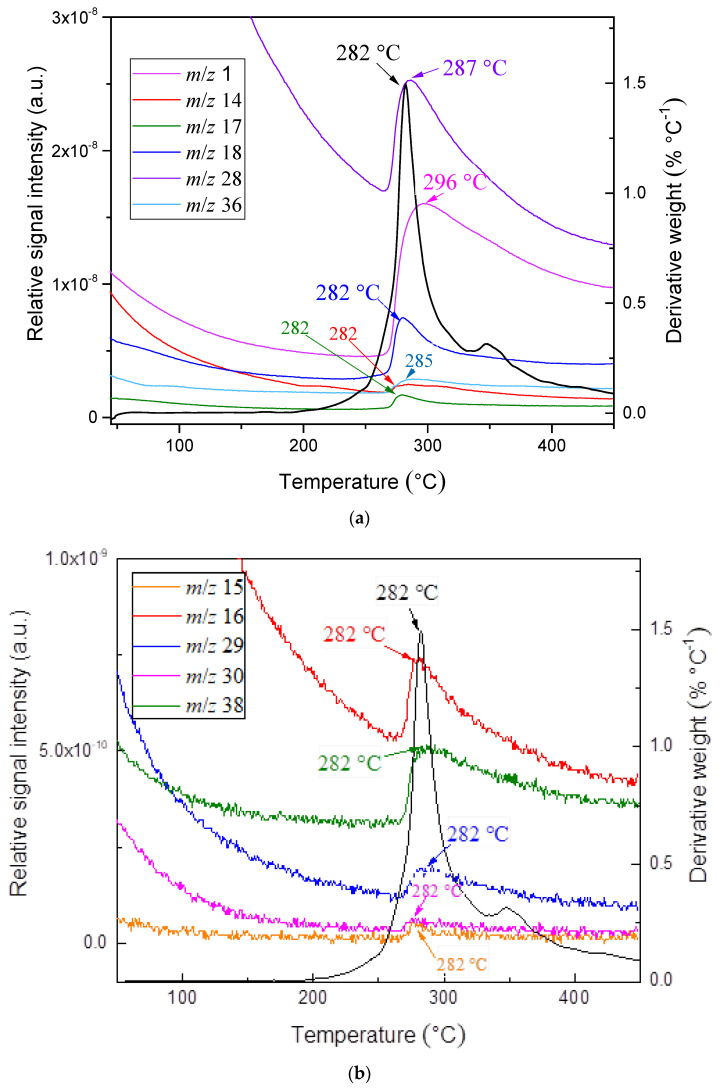
(**a**,**b**) The DTG curves and TG-MS fragment ion intensities for AMB products.

**Figure 4:**
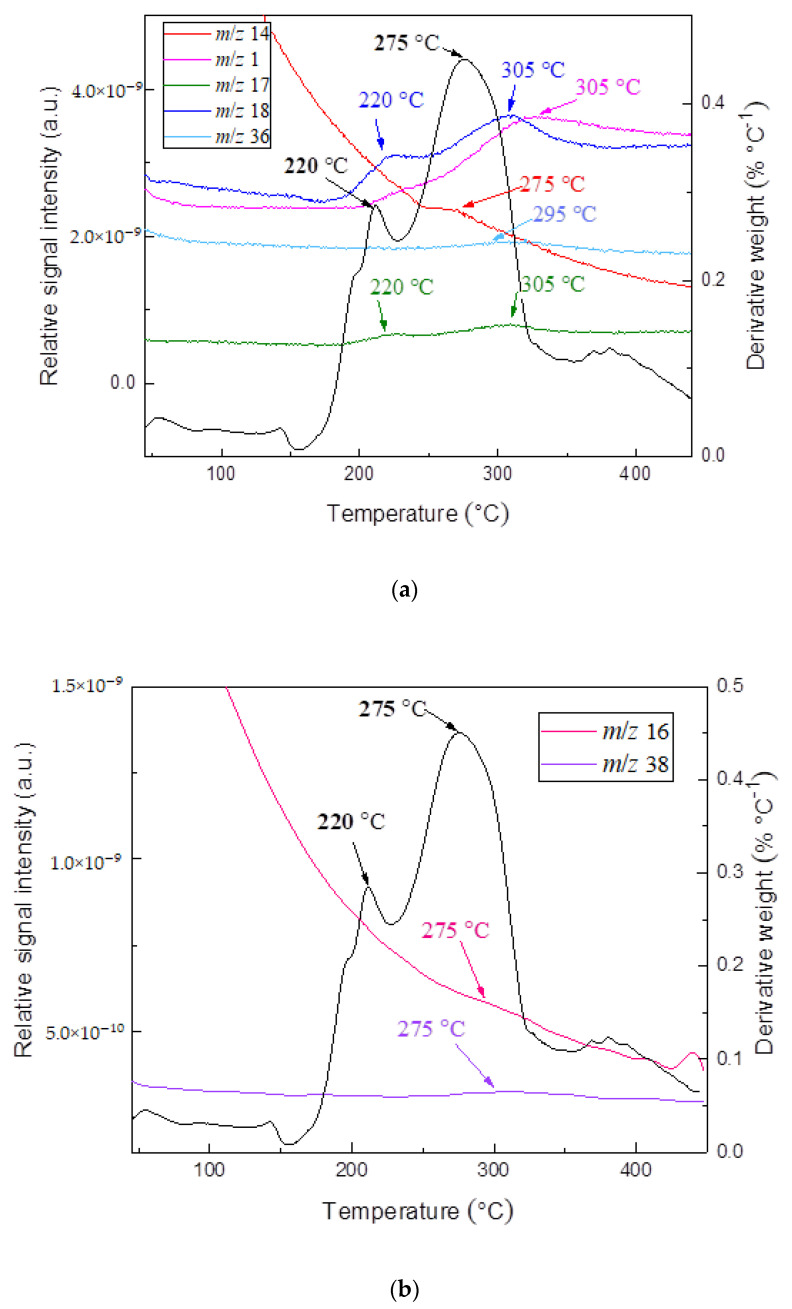
(**a**,**b**) The DTG curves and TG-MS fragment ion intensities for FT products.

In order to obtain a mechanistic explanation of AMB and FT thermal decompositions, TG was coupled with the MS technique. Since there is no information currently available in the literature, the use of the hyphenated technique TG-MS will allow the collection of information about the evolution of the main fragment products throughout the entire degradation process. Figure 3a,b presents DTG curves and TG-MS fragment ion intensities for products formed during thermal decomposition of ambroxol hydrochloride. The DTG curves and TG-MS fragment ion intensities for products formed during thermal decomposition of commercial Flavamed^®^ tablet are presented in Figure 4a,b. Both measurements were conducted in an argon (Ar) atmosphere.

**Figure 5:**
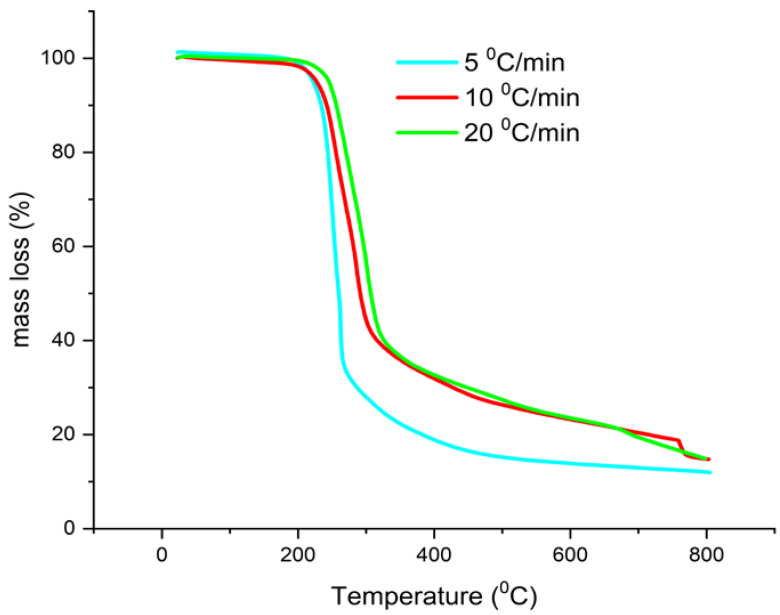
TG curves of AMB in non-isothermal regime in an N_2_ atmosphere.

**Figure 6:**
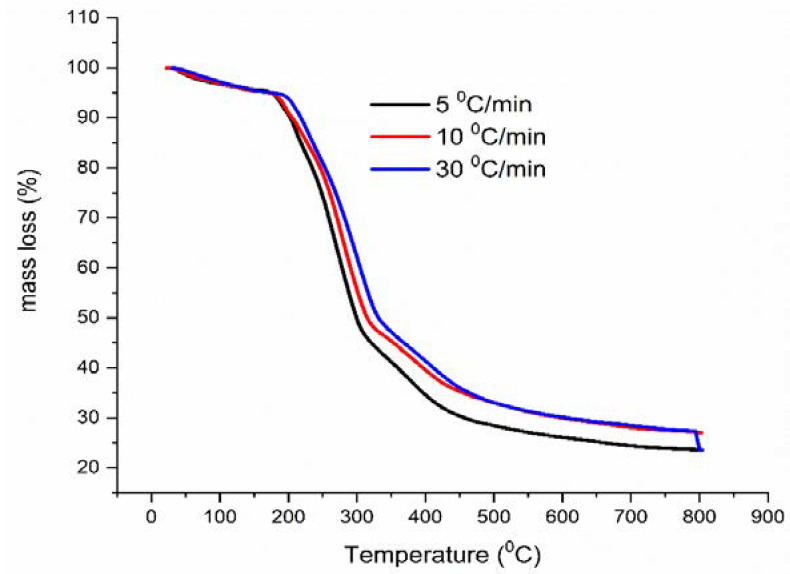
T G curves of FT in non-isothermal regime in an N_2_ atmosphere.

For the evaluation of AMB thermal stability from the kinetic point of view, thermal decomposition of ambroxol hydrochloride and Flavamed^®^ tablet was recorded using multiple heating rates, i.e., non-isothermal conditions as follows: AMB (β = 5, 10, and 20 °C/min) (Figure 5) and FT (β = 5, 10, and 30 °C/min) (Figure 6) in an N_2_ atmosphere. According to the literature data, the stability of ambroxol hydrochloride was mainly studied through the stress degradation approach according to ICH (International Council for Harmonisation of Technical Requirements for Pharmaceuticals for Human Use) guidelines [41,42,43], or thermal stability via drug-drug interactions [25]. It was noted that ambroxol hydrochloride degrades extensively under acid, alkali, and oxidative conditions, considered during storage time [41].

#### Compatibility Study Results

**Figure 7:**
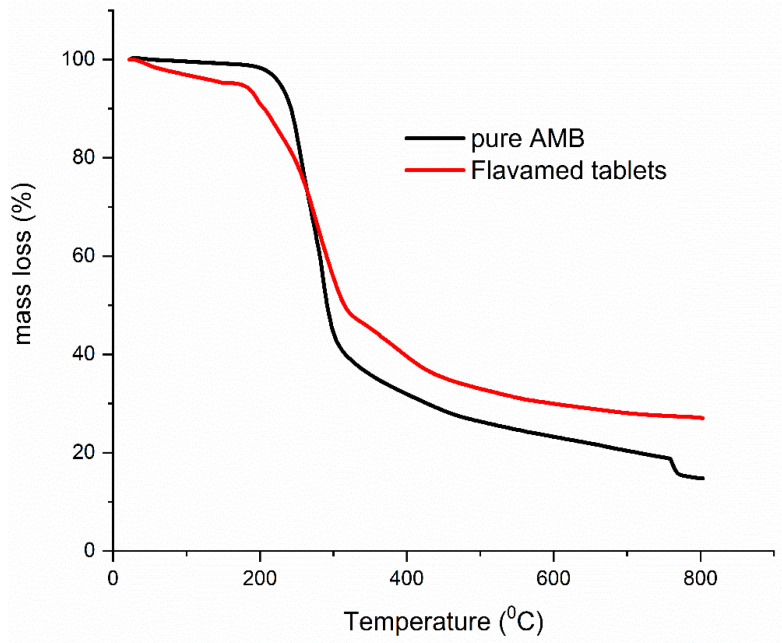
The TG curve of AMB (black line) and FT (red line) at 10 °C·min^−1^ in N_2_ atmosphere.

Excipients in Flavamed^®^ formulation can contain fairly high water content, and they can affect and increase the degradation rate of AMB. Therefore, TG curves of AMB (black line) and FT (red line) were jointly presented in Figure 7 to compare their decomposition paths and TG shape. Evaluating the TG curve of AMB decomposition in the entire temperature region, it seems that the thermal properties of AMB in Flavamed^®^ tablets may suffer from changeable stability. Such findings could be potentially attributed to the previous moisture effect. In addition, it should be noted that lactose monohydrate, one of the excipients in FT formulation, also interacts with moisture-sensitive drugs and affect the stability of the drug [27,44]. Koivisto et al. [45] reported that Mg stearate (excipient as well) properties strongly depend on the moisture content and its hydration state [21]. Literature data confirm that magnesium stearate might absorb moisture during storage under relative humidity greater than approximately 85% [46]. Stanisz et al. [39] pointed out that in some pharmaceutical formulations such as moexipril hydrochloride, Mg stearate should be avoided as excipients. Alternatively, if used, the level of humidity during storage should be minimized. Such findings are crucial, and the influence on the drug stability and thermal properties in a humid atmosphere should be watchful during storage.

**Figure A1:**
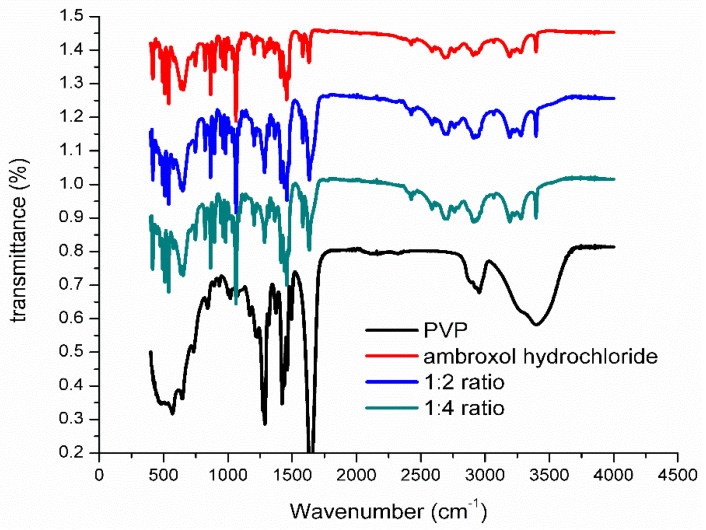
FTIR spectra of pure AMB, PVP, and AMB/PVP mixture in 1:2 and 1:4 ratio.

**Figure A2:**
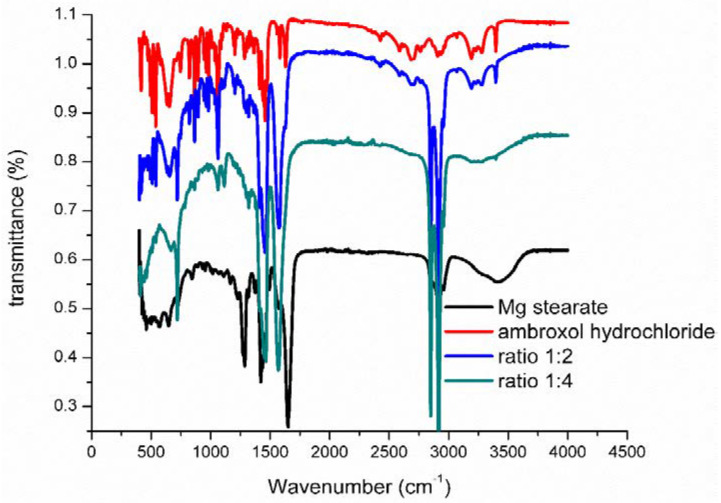
FTIR spectra of pure AMB, Mg stearate, and AMB/Mg stearate mixture in 1:2 and 1:4 ratio.

**Figure A3:**
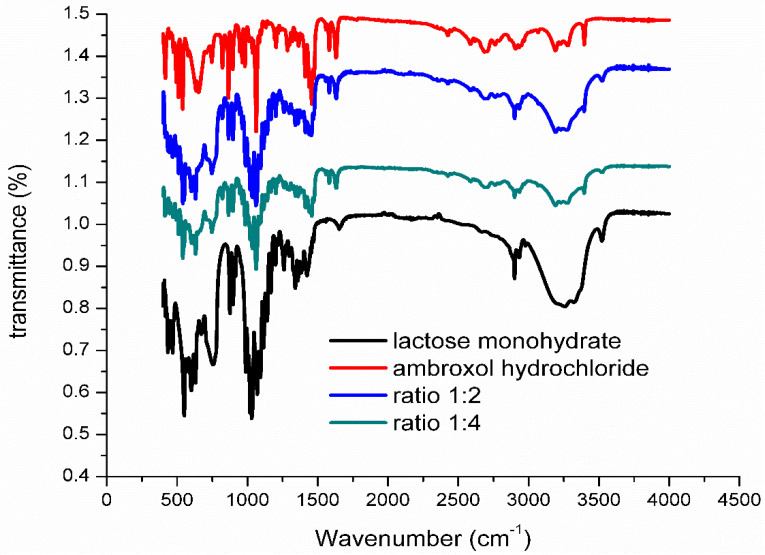
FTIR spectra of pure AMB, lactose monohydrate, and AMB/lactose monohydrate mixture in 1:2 and 1:4 ratio.

**Figure A4:**
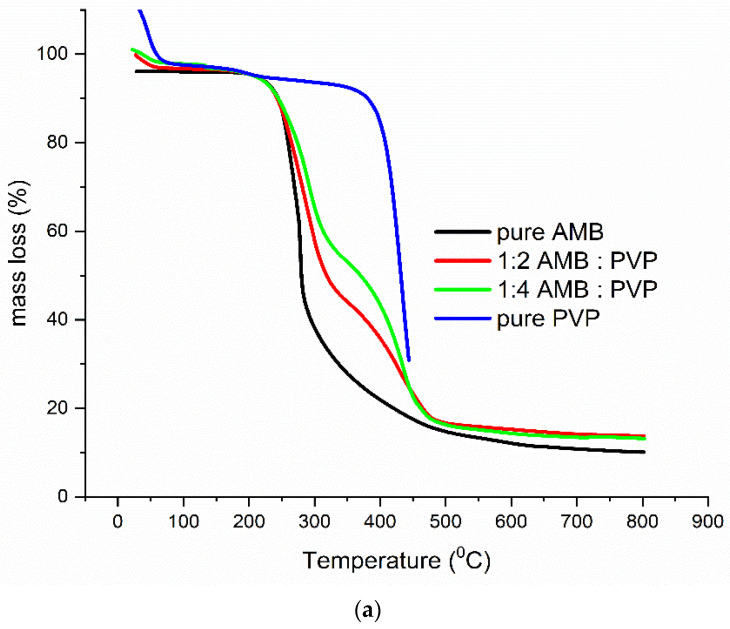

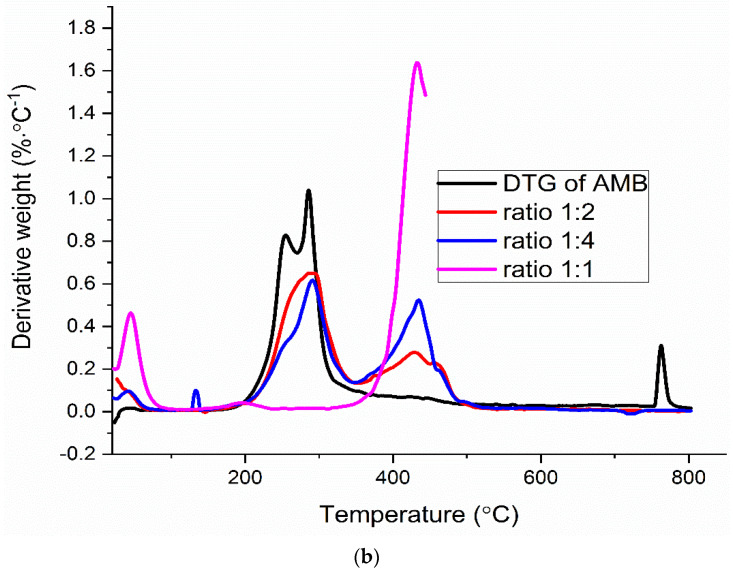
(**a**,**b**)TG/DTG curves of AMB/PVP mixtures.

**Figure A5:**
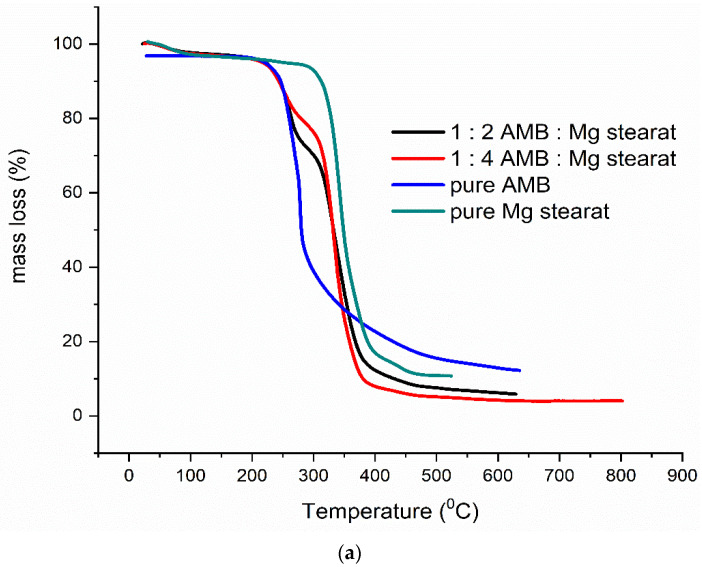

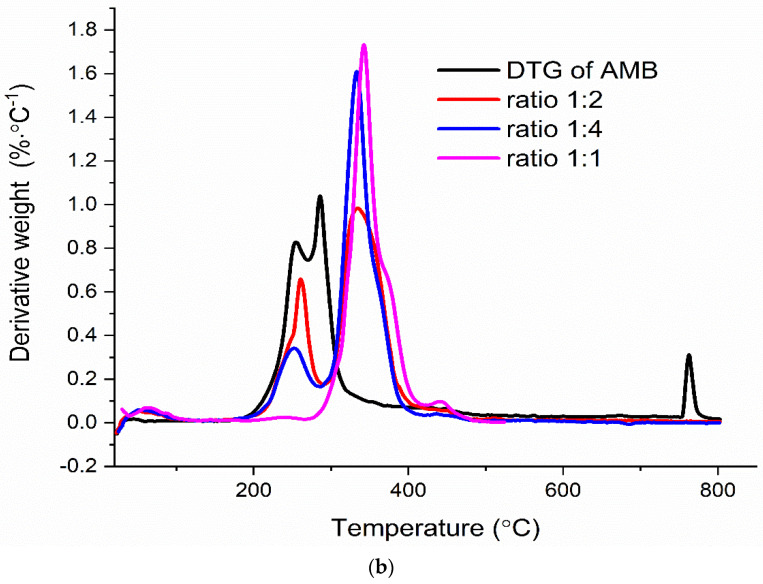
(**a**,**b**) TG/DTG curves of AMB/Mg stearate mixtures.

**Figure A6:**
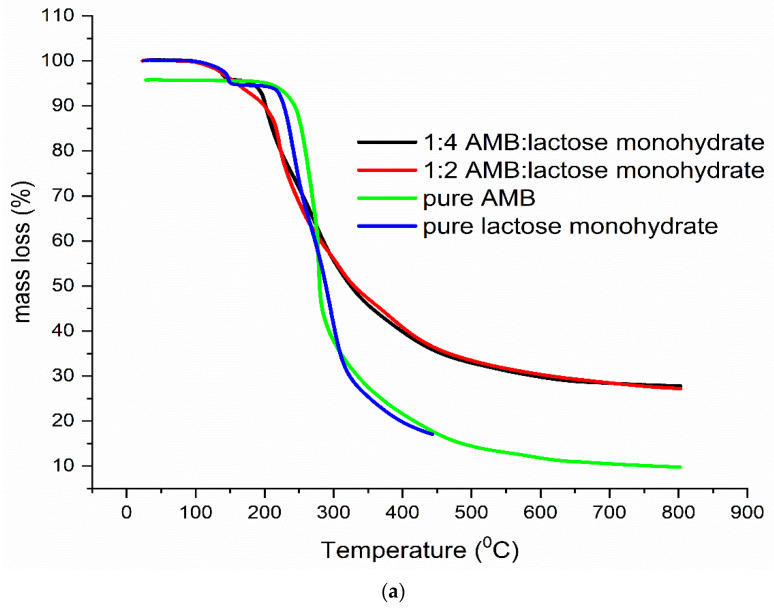

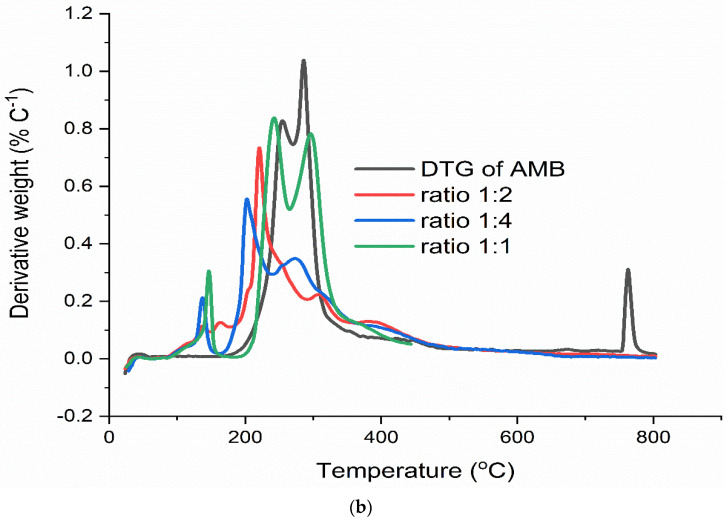
(**a**,**b**) TG/DTG curves of AMB/lactose monohydrate mixtures.

The hypothesis that excipients in the FT formulation contribute to the thermal insult of AMB should also be evaluated. A compatibility test was performed to gain insight into increased/decreased thermal stability information of the active compound. FTIR technique was used to analyze potential interactions between AMB and all excipients (lactose monohydrate, corn starch, powdered cellulose, croscarmellose sodium, PVP, and magnesium stearate) in Flavamed^®^ formulation. Mixtures of AMB and present excipients containing molar ratio 1:1 were formulated and subjected to FTIR analysis (not shown). Upon closer look at FTIR results, Mg stearate, lactose monohydrate, and PVP were observed as potential incompatible components that might have impacted the ambroxol hydrochloride structure. Therefore, to dive deeper into AMB-excipients interactions, we have formulated mixtures containing different molar ratios (1:2 and 1:4) of AMB vs. PVP, Magnesium stearate, and lactose monohydrate. Mixtures 1:2 and 1:4 were subjected to additional FTIR analysis (inserted in Figure A1, Figure A2 and Figure A3—Appendix A part), but also to TG/DTG measurement in N_2_ atmosphere with heating rate, β = 10 °C/min (Figure A4, Figure A5 and Figure A6). It is not uncommonly that excipients can affect the thermal stability of the active compound. Osman et al. noticed that PVP could affect the thermal stability of nifedipine, felodipine, and indomethacin [47]. Brownie et al. worked on and proved the influence of the molecular weight of the polymer and the ratio of vinylpyrrolidone to vinyl acetate in the polymer on ketoprofen solid dispersion [48]. Jelić et al. [49] found that the different amount of PVP in the formulation is significant for the thermal stability of solid dosage forms.

Figure A1, Figure A2 and Figure A3 here (FTIR spectra of AMB/PVP mixture—Figure A1, AMB/Mg stearate mixture—Figure A2 and AMB/lactose monohydrate mixture—Figure A3)—See Appendix A.

Compatibility by TG measurements was evaluated based on changes in TG curves of AMB, pure and in the presence of excipients. TG curves showed undoubtedly that with an increase in PVP (Figure A4) or Mg stearate (Figure A5) content, thermal decomposition curve moves toward higher temperature region, while in case of lactose monohydrate decomposition process is moved toward lower temperature region (Figure A6). Nevertheless, TG curves of PVP and Mg stearate mixtures varied proportionally to excipient content in the mixture, i.e., no incompatibility, but we could not exclude the fact that these excipients affect the thermal stability of active ingredient. This practically means that excipients such as PVP or Mg stearate thermally stabilize, and lactose monohydrate destabilizes AMB in Flavamed^®^ formulation. Interestingly, here the lactose-AMB mixture practically lies in the lower temperature region compared to both ambroxol hydrochloride and pure lactose monohydrate. Thus, it seems that AMB and lactose interactions contribute to their thermal instability mutually. It is important to emphasize that TG data have some disadvantages concerning the compatibility study since TG data provide mass loss % vs. temperature, but on the other hand, TG data offers available information on thermal stability, and taking into consideration DTG graphs, it is very clearly visible that these excipients affect the ambroxol thermal decomposition (Figure A4, Figure A5 and Figure A6).

Figure A4, Figure A5 and Figure A6 here (TG/DTG curves of AMB/PVP mixture—Figure A4, AMB/Mg stearate mixture—Figure A5 and AMB/lactose monohydrate mixture—Figure A6)—See Appendix A.

**Figure 8:**
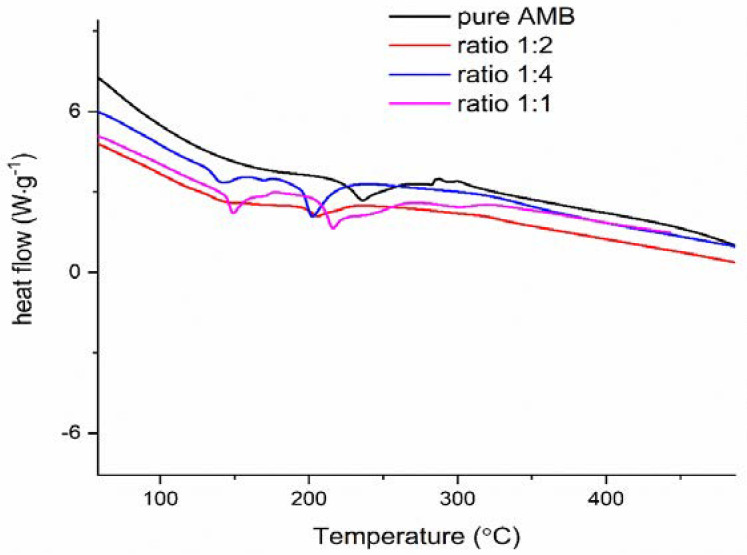
The DSC curves of pure AMB and AMB-lactose mixtures in different ratios.

Further, we exposed compatibility testing to DSC analysis and chemometrics methodology (PCA on FTIR spectra, see Discussion paragraph) to perform compatibility analysis in a systematic exploring way. Figure 8 presents DSC curves of the AMB mixture with lactose in a different ratio.

## 4. Discussion

### 4.1. The Mechanism of Decomposition and Kinetic Analysis of AMB and FT

TG-MS technique used to simultaneously measure the thermal decomposition and ion fragment distribution of each examined compound in real-time showed that shapes of the MS curves followed the shape of the DTG curve, confirming their suitable mutual agreement. Sharp DTG peak corresponds to the high intensity of the MS peaks for the ions with m/z = 18 (H_2_O) and m/z = 17 (OH) (at a temperature of 282 °C). Their intensity ratio m/z = 18 (H_2_O):m/z = 17 (OH) = 5:1, agrees with the National Institute of Standards and Technology (NIST) mass spectral data [50,51,52,53], unambiguously refers to the presence of water. As Figure 3a,b and Figure 4a,b imply, a relatively large amount of H_2_O (m/z = 18) and OH (m/z = 17) is formed, owing to the oxygen atoms present in the structure of the sample. The removal of HCl from ambroxol hydrochloride structure is associated with the HCl (m/z = 36), which releases during heating (T = 285 °C). The advent of that fragment (m/z = 36) is present in the case of Flavamed^®^ tablet in a lower intensity mode and at T = 295 °C. One of the highest intensities for both samples corresponds to the removal of H from structure, m/z = 1. As can be seen from Figure 3a,b, the fragments m/z = 28 and m/z = 14 embedded in this peak around 287 °C: m/z = 14 (CH_2_), while his dimer, m/z = 28 has similar shape (CH_2_CH_2_), with the higher intensity. In the Flavamed^®^ sample, the leaving fragment of the m/z = 14 starts around 275 °C, while his dimer is not presented in Figure 4a,b, because of the noticeable superimposed fragments intensity. The intensity of m/z = 28 is much higher than that of the other MS peaks of these mentioned products. The C-C and C-H bonds break to form free radicals, recombined into small fragments as the temperature increases. Therefore, the most significant portion of m/z = 28 can be attributed to CH_2_CH_2_. As the temperature increase, the C-C and C-H bonds break to form free radicals, which are recombined into small fragments such as m/z = 14 (CH_2_), detected with very low intensities (Figure 3a,b). The reason for the monotonous decrease of m/z = 14 fragment ion intensity with increasing temperature is the gradual release and departure of the fragment. Maximum fragment intensity is expressed at temperatures corresponding to the maximum at the DTG curve and decreases according to the DTG curve. The presence of the small peak at the DTG curve of the Flavamed^®^ tablet sample indicates that at least two overlapping steps occur in this temperature range (220–275 °C). The first sharp DTG corresponds to the high intensity of the MS peaks for the ions with m/z = 18 and m/z = 17 (at a temperature of 220 °C). After that mentioned water molecules releasing process, the same water liberating process occurs at temperature 275 °C. In TG-MS experimentally obtained results, no considerable difference was observed between the ambroxol hydrochloride and commercial Flavamed^®^ tablet (Figure 3a,b and Figure 4a,b). In agreement with the similar modes of thermal decomposition, the mass spectra confirm the similar fragmentation behavior of the two investigated samples. In Table 1, the identification results and normalized ion currents for two investigated compounds in this work are summarized. The gas-phase composition monitored by MS during the thermal decomposition of compounds showed almost the same main decomposition products. The order of magnitude and intensity order of the other released fragments showed a similar decomposition pattern in all investigated samples. There is no significant difference in the mechanistic decomposition of ambroxol hydrochloride pure and Flavamed^®^ tablets.

### 4.2. Kinetics of Ambroxol Hydrochloride and Flavamed^®^ Tablets

#### 4.2.1. Kinetics of the Evaporation Phase

The first stage of the decomposition process for both systems, AMB and FT, is designated as the evaporation phase, in which water loss occurs. It can be observed from Figure 2a,b that the DTG mass event of AMB and Flavamed^®^ tablet system started at about 30 °C and is characterized with a rather insignificant water loss for AMB and considerable surface water loss of the Flavamed^®^ tablets, ΔmFT = 4.98%. The higher water mass loss for FT is somehow expected since some excipients such as lactose monohydrate, PVP, or corn starch, may contain fairly high water content. In addition, this stage embraces the hydrate crystal water, which originates from the dehydration process of lactose monohydrate at approximately 141 °C [54]. The TG data of the evaporation stage in Flavamed^®^ tablets were analyzed by Kinetics2015 software [29]. As previously mentioned, in the theoretical background, two approaches were used: isoconversional and model-fitting approaches. Only the kinetic model that provided the best fit was taken into account, based on the RSS_1_ and RSS_2_. The RSS_1_ and RSS_2_ present the sum of squares of weighted normalized rate residuals and a sum of squares of weighted normalized cumulative residuals. Friedman’s method, isoconversional approach, provided a value of E amounted to 83.119 kJ/mol and A = 4.7329 × 10^12^ s^−1^ with 3.2000 and 0.03565 for RSS_1_ and RSS_2_, respectively. The reaction mechanism that best fits and describes the evaporation stage (dehydration), is the n-th order model with parameters presented as follows: E = 70.83 kJ/mol, A = 1.2722 × 10^9^ s^−1^, n = 3.966, RSS_1_ = 1.58399 and RSS_2_ = 0.89046, respectively. Based on the value of parameter n (the reaction order close to 4), we can conclude that this stage is very complex and involves a series of parallel reactions, where dominates the reaction with a rather high value of the pre-exponential factor (~10^9^ s^−1^). It can be pointed out that the mentioned value of E can be attributed to the lateral diffusion of water molecules to the active sites of some of the structural constituents, which a powdered tablet comprises. Based on the obtained kinetic parameters and the reaction mechanism characteristics, we can conclude that the moisture will significantly impact the kinetics of the ambroxol hydrochloride degradation [55].

**Figure 9:**
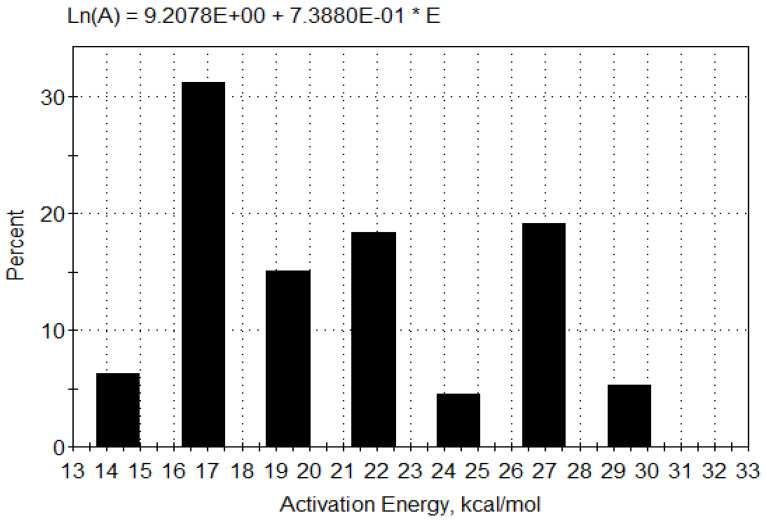
Distribution of activation energy for evaporation stage of FT thermal decomposition process.

Furthermore, we have applied the kinetic option that allows us to obtain the distribution of activation energies over a discrete distribution model, taking into account the unequivocal complexity of this reaction stage. This option gives us the result, which directly connects the values of a pre-exponential factor with values of activation energy through the linear correlation, known as the compensation effect, in the form of the relationship A_i_ = *a*·e*^b^*^·Ei^, where *a* and *b* are constants dependent on the reaction system [56]. Once we determine *a* and *b*, we can estimate the distribution of activation energies uniquely to fit experimental data by a procedure similar to that presented by Vand [57]. Easily determination of parameters *a* and *b* allows us to use the above-mentioned software programs. Figure 9 presents the compensation effect equation (with values of parameters *a* and *b* such as: *a* = 9.2078 s^−1^ and *b* = 7.388 × 10^−1^ mol·kcal^−1^, respectively) along with the distribution of energies. Based on the distribution of E values for the first degradation stage, we can identify the range of activation energies from 59.98 to 123.35 kJ·mol^−1^, having RSS_1_ and RSS_2_ values estimated at 4.4040 and 0.0654, successively. We can see that the most significant percentage of the probability of occurrence (≈31%) takes the reaction, which has a value of E = 70.5 kJ/mol, which is typical for diffusion-controlled processes. Therefore, this could indicate a diffusion-controlled process with the catalytic behavior, where the humidity catalyzes the observed process.

#### 4.2.2. Kinetics of the Ambroxol Hydrochloride Pure and in the Mixture

The second, third, and fourth degradation stages of FT are attributed to the thermal decomposition process of active compound and excipients used in Flavamed^®^ formulation. In the second (“II”) stage, we can notice the existence of deviation on the DTG curve in the temperature interval from 160 to 262 °C, which corresponds well to the decomposition process of lactose monohydrate. Even though it is not sufficiently visible, in this stage (around 170 °C), the re-arrangement process occurs, i.e., lactose monohydrate transforms to anhydrous lactose [54]. The second (160–262 °C) and second (262–318 °C) stages cover the temperature interval when the active compound AMB undergoes melting and thermal decomposition. Most excipients used for Flavamed^®^ formulation are stable up to 300 °C. The last stage (IV stage—318–800 °C) is attributed to the excipients decomposition reactions, including the primary and secondary released products and the carbonization processes. Excipients such as corn starch [40], PVP [3], croscarmellose sodium excipient [58], magnesium stearate and powdered cellulose [21,59], are disintegrated here.

Particular attention should be focused on considering the second and third degradation stages of AMB and FT, in which the melting and decomposition process of AMB occurs. As previously mentioned, the melting temperature of AMB in the powdered tablet was somewhat different in comparison to the pure AMB, which might have an origin in the humidity or present excipients. The second degradation stage, related to the melting of AMB, starts at the approximately 160 °C and ends at approximately 262 °C, while the third stage starts at the approximately 262 °C and ends at 318 °C. However, it must be stressed that a strict separation between the second and third degradation stages does not exist because the third stage has almost continuous outputs from the second stage. Kinetic parameters for the thermal decomposition of AMB (II and III stages) and Flavamed^®^ tablets (II and III stages) are presented in Table 2 and Table 3. Kinetic models that fit both decomposition processes best are mainly in the range of n-th order model, nucleation and growth model, and discrete model. What is significant to mention here is that our observation previously stated concerning humidity and excipients effect in Flavamed^®^ formulation (especially in the II stage) are well reflected through E values of thermal decompositions. E values of Flavamed^®^ decomposition (II stage) are somewhat lowered compared to II stage of AMB, for approximately 20 kJ/mol for each fitted model. Pre-exponential factors have a very similar value, except the Friedman model in Flavamed decomposition, which is relatively high (~10^17^ s^−1^) and favors the high activity and “collision” probability of molecules in a wide range of fraction reacted values. The third stage, even though it showed slightly higher E values in favor of Flavamed^®^ decomposition, is characterized by relatively high E value and pre-exponential factor, A. These high values of E and A refers to expressed thermal stability, which could be attributed to specific interspecies hydrogen and ionic interactions among excipients and the drug [60].

**Figure 10:**
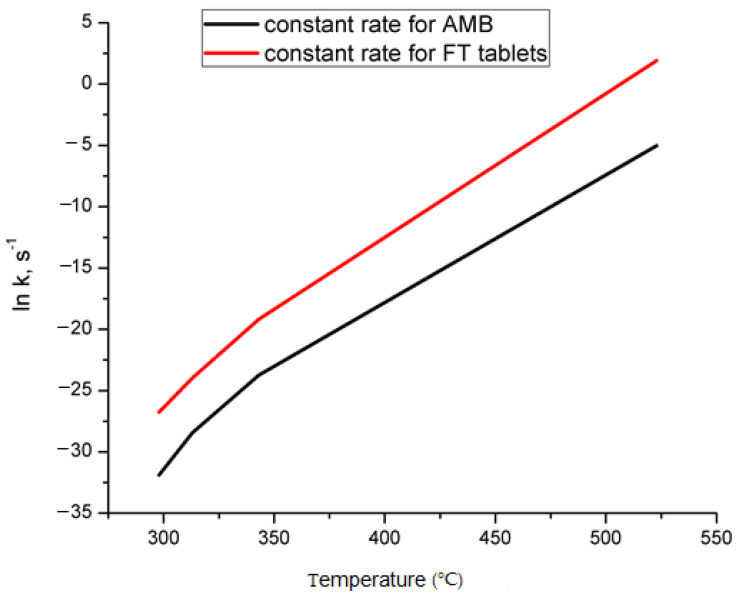
The dependence of constant rate vs. temperature for AMB and FT decomposition mechanism.

Since AMB in Flavamed^®^ tablets seems to be humidity-sensitive drug, we accounted in the stability assessment to calculate the reaction rate “constant” *k_Tj_*_,*h*_, as a function of percent of relative humidity (*h*). The equation Equation (9) is the modification of the Arrhenius equation introduced by Watermann [61,62] as:

(9) $$\begin{array}{l} k_{T_{j},h}=A\cdot e^{B\cdot h-\frac{E}{RT_{j}}}\\ \ln k_{T_{j}}{{}_{,}}_{h}{}_{}=\ln A+Bh-E/RT_{j} \end{array}$$

where *h* is the relative humidity (%), while *B* is the humidity sensitivity factor (%RH^−1^). The values of *k_Tj_*_,*h*_ were calculated at each value of *T_j_* following the data obtained from the manufacturer in the case of active drug substance, which is directly related to the values *h* and *B*, and they were as follows: *h* = 75% and B = 0.05% RH^−1^. Values of constant rate for pure AMB and FT tablets up to 250 °C are presented in Figure 10. The decomposition process of FT tablets is favored, and it occurs fast. At the same time, the dependence of *k* vs. *T* shows the exact mechanism of degradation for AMB and FT formulation, i.e., the n-th order.

The stability parameter denoted as the “shelf-life” was calculated using the data that best fitted the decomposition mechanism of AMB and FT tablets. Such an approach in non-isothermal thermogravimetric data for shelf-life prediction of drugs was recently used by Calvino et al. [12]. Shelf-life data are presented in Table 4. Shelf-life data of AMB refers that AMB is not a heat-sensitive drug under normal storage conditions and does not require special care during the storage period. Still, undoubtedly AMB thermal stability in Flavamed^®^ tablets is altered. The data for the shelf-life has a high magnitude rather. However, one should consider that thermal decomposition of pure AMB and Flavamed^®^ formulation was conducted in a neutral atmosphere of N_2,_ which can contribute to process delay and parameters such as oxygen and light exposure, different percentage of humidity were omitted and observed.

This paper introduces one more quantity, which may impact the apparent activation energy of the considered degradation stage, and this factor is known as the activation entropy (Δ*S*^#^). Using entropy as a guiding tool for drug development has been recently come into focus [63]. The entropy could be calculated based on the following equation Equation (10) [64]:

(10) $$\Delta S^{\#}=R\left(\ln A-\ln T_{\max}\right)-205.86,\;$$

where *T_max_* represents the maximum (peak) temperature values at a specific heating rate. Values for *T_max_* at *β* = 10 °C/min were extracted from the kinetic software. The term 205.86 is a “solid” constant, which is numerically derived. Values of activation entropy are included in Table 5.

The positive value of entropy reflects increased disorder in the system under surveillance and vice versa. We can see that the thermal decomposition process of Flavamed^®^ tablet, having a high positive value, is a source of entropy. It is worth mentioning that the release of structured water molecules promotes positive entropy due to an increase in the system’s disorder, which follows our findings for the start-up of Flavamed^®^ tablets decompositions. Low or negative values of Δ*S*^#^ are favorable for thermal decompositions that suffer from a decreased number of collisions, i.e., the system disorder is decreased, and such thermal processes can be considered slow.

##### Compatibility Results

The compatibility study was performed using FTIR and TG-DSC techniques. The FTIR spectra were analyzed by PCA. The principal infra-red absorption peaks of pure AMB show characteristic peaks belonging at 647.42, 1064, 1460, 1627.05, and 3392.78 cm^−1^ corresponding to C-Br stretching, C=C bending, C-H bending, N-H stretching, and O-H starching, respectively [65]. The leading absorption bands of AMB and excipients mixture showed the same positions and degree of sharpness in the PVP mixture. In contrast, in the Mg stearate and lactose mixture, there is some deviation (less intensity and position changed) from the ambroxol spectrum, especially in parts 600–800 and 1050–1067 cm^−1^.

**Figure 11:**
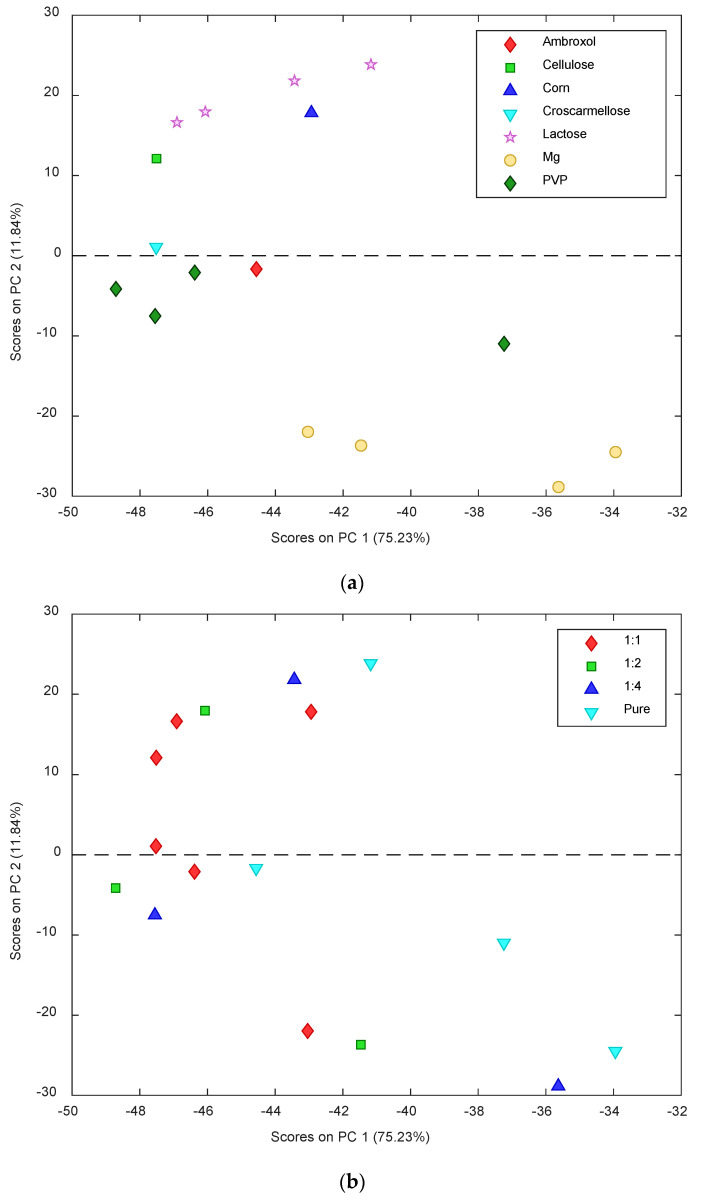

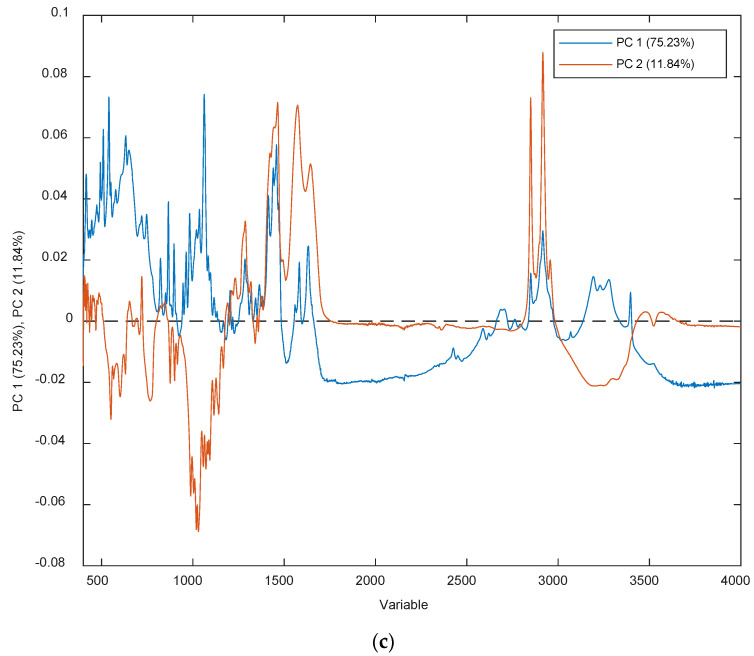
Principal component analysis (**a**) scores plot with samples distinguished as a function of the components; (**b**) scores plot with samples distinguished as a function of the ratio of the components; (**c**) loadings plot.

Principal components analysis (PCA) was employed to explore the collected data to extract the most interesting information from the samples analyzed by IR methodology [66,67,68,69,70]. PCA can be a valuable supplementary tool for assessing compatibilities features and their interpretation [71]. PCA was performed to investigate the available IR data involving the 16 samples containing the pure compounds of AMB, PVP, lactose monohydrate, and Mg stearate, the 1:1 mixtures of AMB with cellulose, croscarmellose, corn, PVP, lactose monohydrate, and Mg stearate, and the 1:2 and 1:4 mixture of AMB with PVP, lactose monohydrate and Mg stearate. The PCA consisted of three principal components describing an overall 87% of the variance (75% of explained variance for the first PC, 12% for the second PC). The samples are arranged into three main clusters, as seen from the first scores plot (Figure 11a). The first cluster shows high values of PC2, containing the samples of pure lactose monohydrate, lactose mixed with AMB, corn mixed with AMB, and cellulose mixed with AMB. In particular, the loadings plot (Figure 11c) indicates that the wavelengths showing the highest positive values of PC2 are those in the ranges of 2900–3100 cm^−1^ and 1500–1800 cm^−1^. On the other hand, a second cluster containing the samples of pure Mg stearate, Mg stearate mixed with AMB, and, partially, the pure PVP compound, shows low values of PC2, together with a high value of PC1. According to the loadings plot, these samples are particularly described by the wavelengths within the range of 500–1200 cm^−1^. Finally, no significant wavelengths were observed for the third cluster composed of pure AMB, the AMB mixed with croscarmellose, and the mixtures containing PVP and AMB.

Therefore, it seems that FTIR analysis showed an absence of physical and chemical interactions between AMB and PVP, while for Mg stearate and lactose monohydrate incompatibility is noticeable. It can be pointed out that increasing the content of magnesium stearate increases the effective rate constant for reaction participation with HCl, which suggests that the products of lubricant decay catalyze the splitting-off of HCl. In the course of the reaction of magnesium stearate with eliminated HCl, stearic acid can be formed. The stearic acid can take part in one of the reaction cycles, which have a catalytic nature during the degradation process of AMB, in the framework of dehydrochlorination [72]. In the case of lactose monohydrate, the Maillard reaction occurs [73]. According to DSC graphs, all three components enrolled in the mixtures showed changes. For Mg stearate, the prominent melting peak of AMB is well pronounced, but peaks at 282 and 296 °C corresponding to m/z = 18 and m/z = 36 are missing. In the case of lactose mixture melting, the peak is moved to 30 °C left and relatively broader. A similar observation we have for peaks of Mg stearate mixture, also. For the PVP mixture, the melting peak of AMB is practically invisible; no heat change of AMB during its melting is recorded in the presence of PVP.

It is worth mentioning that sometimes interaction (denoted in this work as incompatibilities) between active compounds and excipients can be favorable. For example, in the case of PVP, such interactions can contribute to better solubility when it comes to poorly soluble drugs [74].

The compatibility results obtained by FTIR, TG-DTG, DSC, and chemometrics analyses are summarized in Table 6.

## 5. Conclusions

The ambroxol hydrochloride is not a heat-sensitive drug, but moisture and crystal water could promote faster degradation of ambroxol hydrochloride. During formulation studies, special attention for excipients present in the formulation mixture should be considered. Compatibility study was performed using FTIR and TGA-DSC analysis. Moreover, the FTIR spectra were analyzed by PCA. The data analysis highlights the incompatibility of ambroxol with the Mg stearate and lactose monohydrate. In Flavamed^®^ tablets, it was proven that excipients such as PVP, Mg stearate, and lactose monohydrate could affect thermal stability and mechanism of decomposition. This is a consequence of the ambroxol hydrochloride structure, in which HCl is being liberated, providing an acid environment that can be very beneficial for different reactions such as hydrolysis. Consequently, the decomposition process proved to be a multistep and very complex one, which is best described with the n-th order model with the following kinetic parameters:

pure AMB kinetics

II stage—E = 155 kJ·mol^−1^, A = 2.0 × 10^13^ s^−1^, n = 0.6.

III stage—E = 199 kJ·mol^−1^, A = 4.5 × 10^16^ s^−1^, n = 1.57.

Flavamed^®^ tablets kinetics

II stage—E = 143 kJ·mol^−1^, A = 2.8 × 10^13^ s^−1^, n = 1.2.

III stage—E = 207 kJ·mol^−1^, A = 2.0 × 10^16^ s^−1^, n = 2.0.

Based on the decomposition model, calculated shelf-life once again confirmed AMB thermal stability issues when present in the mixture.

Results obtained with this elaborate study, especially the kinetics one, provide potential guidelines and future perspectives for preparing successful and stable formulations of ambroxol-containing drugs.

## Figures and Tables

**Table 1:** Summarized identification results and normalized ion currents for investigated samples of ambroxol hydrochloride and commercial Flavamed^®^ tablet.

m/z Fragment	Normalized Ion Current for Compounds	
	Ambroxol Hydrochloride	Commercial Flavamed Tablet
1	1.5 × 10^−8^	3.5 × 10^−9^
14	2.5 × 10^−9^	2 × 10^−9^
15	5.5 × 10^−11^	not available
16	7.5 × 10^−10^	6 × 10^−10^
17	2.0 × 10^−9^	1 × 10^−9^
18	8.0 × 10^−9^	4 × 10^−9^
28	2.5 × 10^−8^	not available
29	2 × 10^−10^	not available
30	6 × 10^−11^	not available
36	3 × 10^−9^	2 × 10^−9^
38	5 × 10^−10^	3 × 10^−10^

**Table 2:** The isoconversional and model-fitting kinetic analysis of AMB thermal decomposition: II stage (upper/first table) and III stage (down/second table).

II Stage of Pure AMB Decomposition					
Friedman model					
*A*, 1/s	*E*, kJ/mol	*n*		RSS_1_	RSS_2_
1.794 × 10^14^	164.71	1		1.8128	0.3146
n-th order					
*A*, 1/s	*E*, kJ/mol	*n*		RSS_1_	RSS_2_
2.005 × 10^13^	155.845	0.666		3.0604	0.2029
Nucleation and growth					
*A*, 1/s	*E*, kJ/mol	*n*	*m*	RSS_1_	RSS_2_
9.311 × 10^13^	161.74	0.881	0.0349	3.4053	0.2256
Discrete distribution model					
*A*, 1/s	*E*, kJ/mol			RSS_1_	RSS_2_
2.420 × 10^15^	175.803 (98.54%)			4.6647	0.55329
III Stage of Pure AMB Decomposition					
Friedman model					
*A*, 1/s	*E*, kJ/mol	*n*		RSS_1_	RSS_2_
5.62 × 10^18^	202.559	1		4.0662	0.3155
n-th order					
*A*, 1/s	*E*, kJ/mol	*n*		RSS_1_	RSS_2_
4.553 × 10^16^	199.66	1.5735		3.9335	0.38758
Nucleation and growth					
*A*, 1/s	*E*, kJ/mol	*n*	*m*	RSS_1_	RSS_2_
1.6148 × 10^19^	221.165	2.747	0.46121	9.4676	0.65075
Discrete distribution model					
*A*, 1/s	*E*, kJ/mol			RSS_1_	RSS_2_
1.2604 × 10^17^	209.29 (50.40%)	200.9 < ΔE < 213.47		3.4465	0.36096

**Table 3:** The isoconversional and model-fitting kinetic analysis of FT formulation thermal decomposition: II stage (upper/first table) and III stage (down/second table).

II stage of Flavamed^®^ Decomposition					
Friedman model					
*A*, 1/s	*E*, kJ/mol	*n*		RSS_1_	RSS_2_
5.446 × 10^19^	150.05	1		4.104	0.05809
n-th order					
*A*, 1/s	*E*, kJ/mol	*n*		RSS_1_	RSS_2_
2.8245 × 10^13^	143.38	1.2567		6.7365	0.16284
Nucleation and growth					
*A*, 1/s	*E*, kJ/mol	*n*	*m*	RSS_1_	RSS_2_
1.6334 × 10^14^	148.79	1.735	0.136	9.1320	0.22973
Discrete distribution model					
*A*, 1/s	*E*, kJ/mol			RSS_1_	RSS_2_
1.1935 × 10^14^	150.68 (86.15%)	142.31 (13.85)		4.6074	0.11721
III stage of Flavamed^®^ decomposition					
Friedman model					
*A*, 1/s	*E*, kJ/mol	*n*		RSS_1_	RSS_2_
2.855 × 10^17^	207.51	1		2.1234	0.00135
n-th order					
*A*, 1/s	*E*, kJ/mol	*n*		RSS_1_	RSS_2_
2.0945 × 10^16^	207.93	2.0994		8.56969	0.20434
Discrete distribution model					
*A*, 1/s	*E*, kJ/mol			RSS_1_	RSS_2_
1.2817 × 10^17^	209.29 (50.84%)	192.54 < ΔE < 209.29		0.81406	0.00747

**Table 4:** The shelf-life for pure AMB and in Flavamed^®^ formulation.

Temperature/°C	Shelf-Life of AMB	Shelf-Life of Flavamed Tablets
25	233,324 years	1366 years
40	12,089 years	83.3 years
70	66.7 years	8.82 month
250	15.62 s	0.0155 s

**Table 5:** The value of Δ*S*^#^, J·mol^−1^·K^−1^ for the II and III stages of AMB and FT decomposition process.

	AMB II Stage	AMB III Stage	FT II Stage	FT III Stage
Δ*S*^#^/J·mol^−1^·K^−1^	14.79	100.5	120.6	75.76

**Table 6:** The comparative analysis of compatibility study performed on AMB and FT systems.

	AMB/Lactose	AMB/PVP	AMB/Mg Stearate
FTIR-PCA	✓	*	✓
TG	✓	*	*
DSC	✓	✓	✓

Tick sign (✓) stands for incompatibility; star sign (*) denotes compatibility.
